# Sustainability for humans and the humane from a pediatric point of view

**DOI:** 10.1192/j.eurpsy.2022.1759

**Published:** 2022-09-01

**Authors:** Å. Victorin

**Affiliations:** Gothenburg Public health, School Medicine, Hovås, Sweden

**Keywords:** sustainability, mental health, prevention, Adolescents

## Abstract

**Introduction:**

We need to live in harmony with our lifestyle rhythms to stay healthy. A problem in our time is that technical devices have no respect for rhythm.If we get caught up in the technique and start neglecting our natural body needs such as sleep, eat and exercise – it will affect our health negatively. Today, children have increasing problems with mental health. When analyzing the problem we find rhythmical problems, often associated to technology. Being a parent in our time is hard. Time has come for us to take active care of our natural rhythms, to stay healthy.

**Objectives:**

Increasing mental health problems among the young is a global issue in the industrialized world. We see a connection between digitization, the intro of smartphones 2007 and the increase of anxiety, depression and melt downs in children who are left with to little adult guidance. Their screen time becomes to long leading to impaired health due to long sedentary time. The result is not enough physical activity, obesity, introversy because of lack of IRL social contact etc. The problems are well known but why don´t we talk more about them and help our children to deal with it?

**Methods:**

Read any statistics about mental helath among the young in the industrialized countries.

**Results:**

While studying the statistics in Sweden over time the results of the bad sustainability of living are clear.

**Conclusions:**

The adult world needs a wake up call which I will give in my oral presentation. I will also present proposals of solution.

**Disclosure:**

No significant relationships.

